# Sustainability in Health care by Allocating Resources Effectively (SHARE) 9: conceptualising disinvestment in the local healthcare setting

**DOI:** 10.1186/s12913-017-2507-6

**Published:** 2017-09-08

**Authors:** Claire Harris, Sally Green, Wayne Ramsey, Kelly Allen, Richard King

**Affiliations:** 1 School of Public Health and Preventive Medicine, Monash University, Melbourne, Australia; 2 Centre for Clinical Effectiveness, Monash Health, Melbourne, Australia; 3 Medical Services and Quality, Monash Health, Melbourne, Australia; 4 Medicine Program, Monash Health, Melbourne, Australia

**Keywords:** Disinvestment, Decommissioning, De-adopt, De-implement, Resource allocation, Reinvestment, Reallocation, Rationing, Prioritisation, Decision-making

## Abstract

**Background:**

This is the ninth in a series of papers reporting a program of Sustainability in Health care by Allocating Resources Effectively (SHARE) in a local healthcare setting. The disinvestment literature has broadened considerably over the past decade; however there is a significant gap regarding systematic, integrated, organisation-wide approaches. This debate paper presents a discussion of the conceptual aspects of disinvestment from the local perspective.

**Discussion:**

Four themes are discussed: Terminology and concepts, Motivation and purpose, Relationships with other healthcare improvement paradigms, and Challenges to disinvestment.

There are multiple definitions for disinvestment, multiple concepts underpin the definitions and multiple alternative terms convey these concepts; some definitions overlap and some are mutually exclusive; and there are systematic discrepancies in use between the research and practice settings. Many authors suggest that the term ‘disinvestment’ should be avoided due to perceived negative connotations and propose that the concept be considered alongside investment in the context of all resource allocation decisions and approached from the perspective of optimising health care. This may provide motivation for change, reduce disincentives and avoid some of the ethical dilemmas inherent in other disinvestment approaches.

The impetus and rationale for disinvestment activities are likely to affect all aspects of the process from identification and prioritisation through to implementation and evaluation but have not been widely discussed.

A need for mechanisms, frameworks, methods and tools for disinvestment is reported. However there are several health improvement paradigms with mature frameworks and validated methods and tools that are widely-used and well-accepted in local health services that already undertake disinvestment-type activities and could be expanded and built upon.

The nature of disinvestment brings some particular challenges for policy-makers, managers, health professionals and researchers.

There is little evidence of successful implementation of ‘disinvestment’ projects in the local setting, however initiatives to remove or replace technologies and practices have been successfully achieved through evidence-based practice, quality and safety activities, and health service improvement programs.

**Conclusions:**

These findings suggest that the construct of ‘disinvestment’ may be problematic at the local level. A new definition and two potential approaches to disinvestment are proposed to stimulate further research and discussion.

**Electronic supplementary material:**

The online version of this article (doi:10.1186/s12913-017-2507-6) contains supplementary material, which is available to authorized users.

## About SHARE

*This is the ninth in a series of papers reporting Sustainability in Health care by Allocating Resources Effectively (SHARE). The SHARE Program is an investigation of concepts, opportunities, methods and implications for evidence-based investment and disinvestment in health technologies and clinical practices in a local healthcare setting. The papers in this series are targeted at clinicians, managers, policy makers, health service researchers and implementation scientists working in this context. This paper discusses current research and debate in disinvestment as it applies in local healthcare settings*.

## Background

There are many challenges to the sustainability of healthcare services. Ageing populations and the rising prevalence of chronic diseases, increasing use of new and existing health technologies, duplication and gaps in service delivery from poorly coordinated care, ineffective practices, systemic waste and external economic pressures all threaten the ability to maintain health services at optimal standards [1–8].

The primary focus of health care should be on optimising patient outcomes, but without due consideration of value for money the system will not be sustainable [9, 10]. Rigorous processes have been established to ensure that new health technologies and clinical practices (TCPs) are safe, effective and cost-effective and that their introduction will result in better health outcomes [11–15]. However many TCPs in current use were not evaluated rigorously prior to their introduction and would not meet contemporary standards [16]; some were commenced prior to establishment of these processes or the processes were not applied [11, 13, 17, 18]; some were implemented based on early evidence and the initial promising findings were reversed in subsequent studies [19–21]; the effectiveness and cost- effectiveness of many is unknown [19, 22] and others which have been demonstrated to be effective and cost-effective are used inappropriately or alternatives with greater benefits are available [23, 24]. The number of patients receiving potentially unsafe or ineffective care is worryingly high. In a recent sample of US Medicare beneficiaries, 25–42% received at least one intervention considered to be ‘low value’ [25].

Debate and research have turned towards opportunities to reduce costs and maximise outcomes by removing, reducing or restricting these sub-optimal practices and the concept of disinvestment has emerged.

The early disinvestment literature was focused on two main areas: research guided by health economic principles to disinvest specific TCPs in a local setting and broader discussion focused on central policy-making and the role of national agencies to inform decisions [26–28]. More recently, additional topics and perspectives have been addressed in commentary and editorials [9, 29–36] and national and international approaches have been explored in discussion papers and reports [10, 37–44]. Systematic reviews have been conducted to inform disinvestment projects on specific conditions or diseases [45–47] and authors of systematic reviews addressing standard clinical questions are now routinely commenting on practices of ‘low value’ in their topic area [48]. Wider generic questions about the context, settings, systems, processes and principles for disinvestment have been addressed in systematic reviews [26, 47, 49–56] and other studies [13, 23, 39, 57–65]. Lists identifying ‘low value’ practices for potential disinvestment have been produced for clinicians and policy makers [19, 21, 59, 66–72] and have subsequently generated further debate about their validity and applicability [73–79].

Although the research and debate has broadened considerably, there remains a significant gap in the literature regarding systematic, integrated approaches to disinvestment. In particular, there is little information to guide healthcare networks or individual facilities in how they might take an organisation-wide approach to disinvestment [26, 37, 39, 45, 50, 51, 58, 60, 80, 81].

The ‘Sustainability in Health care by Allocating Resources Effectively’ (SHARE) Program was an organisation-wide, systematic, integrated, transparent, evidence-based approach taken by one Australian health service to address these issues at the local level. Monash Health (previously Southern Health) is a public network of six acute hospitals, subacute and rehabilitation services, mental health and community health services, and residential aged care [31]. Australian public hospitals operate under a state-allocated activity-based fixed-budget model of financing [32]. Staff are salaried and services are provided free of charge. An overview of the SHARE Program, further details about Monash Health and a guide to the SHARE publications are provided in the first paper in this series [82] and a summary of the outcomes is included in the final paper [83].

This review of the literature was initially commenced to form the background to the first paper in the SHARE series. However it became obvious that in order to address the gaps in knowledge and understanding about systematic approaches to disinvestment at the local level, the review would be improved by inclusion of the findings of the SHARE Program. The logical extension of this was to place the review after the other papers in the SHARE series.

The substantial body of literature available was too large for a single publication. As multiple themes emerged, it was clear that they could be readily divided into topics related to either conceptualisation or operationalisation of disinvestment. This paper focuses on the conceptual elements of disinvestment at the local health service level. It is a companion to the tenth paper of the SHARE series which considers the disinvestment literature from an operational perspective [84]. The contents of both reviews are summarised in Table 1.

**Table 1:** Contents of the literature reviews

SHARE Paper 9. Conceptual perspective
▪ Terminology and concepts – Health technologies – Disinvestment – Resource allocation – Optimising health care – Reinvestment ▪ Motivation and purpose – Impetus for disinvestment – Rationale for disinvestment ▪ Relationships with other healthcare improvement paradigms – Evidence based health care – Quality improvement – System redesign – Health economic approaches ▪ Challenges ▪ New approach to disinvestment
SHARE Paper 10. Operational perspective
▪ Existing theories, frameworks and models ▪ New framework ▪ Program – Principles of decision-making – Settings and opportunities – Prompts and triggers – Steps in the disinvestment process ▪ Projects ▪ Research ▪ Methods and tools – Identification of opportunities – Prioritisation and Decision-making – Development of a proposal – Implementation – Monitoring, Evaluation and Reporting – Reinvestment – Dissemination and Diffusion – Maintenance ▪ Barriers and enablers

The reviews are presented as debate papers to discuss the disinvestment literature from the local healthcare perspective but, since the arguments are based on the findings of a literature review, readers need to have confidence that the process was rigorous and as comprehensive as possible. Although undertaken systematically, this was not a systematic review. It is impossible to be absolutely comprehensive in ascertaining all the relevant literature on disinvestment for two main reasons. Firstly, there is no general agreement about use of the term ‘disinvestment’, it is used to convey multiple concepts, and there are many other terms used to convey the same range of concepts. Secondly, the aims, activities and outcomes of disinvestment initiatives are replicated in research and practice in other healthcare paradigms and published in various bodies of literature. Extensive searches were undertaken to ensure as much as possible that the discussion correctly reflects the literature. The methods of the literature review are included in Additional file 1.

Four themes are discussed in this paper: Terminology and concepts, Motivation and purpose, Relationships with other healthcare improvement paradigms, and Challenges to disinvestment. Findings from the literature and experiences in the SHARE Program suggest that these themes have specific relevance to local healthcare services; in particular, they highlight the difficulties in introducing disinvestment initiatives in this context.

The reason for inclusion of each theme is explained and the discussion is structured to present current thinking from the literature; experiences from the SHARE program; and implications for policy, practice and research in the local healthcare setting for each theme.

In exploring these themes, ways to address some of the problematic issues emerged and a new definition and potential approaches to disinvestment are proposed.

### Aims

The aims of this debate paper are to discuss the current literature on disinvestment from a conceptual perspective, consider the implications for local healthcare settings, and propose a new definition and two potential approaches to disinvestment in this context to stimulate further research and discussion.

## 1. Terminology and concepts

There are multiple definitions for disinvestment, a lack of common understanding of the reasons or objectives that underpin the concept, and disparity in use of the term between the research and practice settings. These shortcomings create difficulties in the interpretation of disinvestment and establishment of a systematic approach in the local healthcare setting.

### 1.1 Health technologies

#### Definition

Most discussion about disinvestment is centred on the use of health technologies; however the term ‘health technologies’ is used with a range of meanings. Definitions of ‘health technologies’ in the literature can be characterised in four groups (Table 2). The first is broad and includes every element of healthcare delivery [22, 61, 85, 86]. The second uses only a selection of these elements [12, 42, 87–90]. The third does not use a specific definition but suggests that health technologies are separate from other elements by including ‘health technologies’ within a list of selected items [27, 45, 49, 51, 53, 91–93]. The fourth is narrow and reflects only medical products and devices [23, 26, 38, 39, 50, 87, 94–96]. Many studies involving health service stakeholders in discussions about health technologies do not specify a definition but choose medical devices or diagnostic equipment as their examples [41, 45, 88, 97].

**Table 2:** Examples of use of the term ‘health technologies’

Scope	Definition or use
Definition encompasses all elements across the spectrum of healthcare delivery and management	“Drugs, diagnostic tests, including indicators and reagents, devices, equipment and supplies, medical and surgical procedures, support systems, and organizational and managerial systems used across the spectrum of health care” [85]
Definition based on a selection of elements from the extensive list above	“Drugs, devices, procedures and screening” [87], “drugs, devices and procedures” [12, 90], “devices, diagnostics and digital technologies” [89], “Pharmaceuticals, devices, diagnostic tests and interventional procedures” [88], “drugs, diagnostic and procedural interventions” [42]
No definition, but wording suggests that health technologies are separate from other elements	“Health care practices, procedures, technologies and pharmaceuticals” [49, 91, 93], “health technology, drug or intervention” [51], “Technologies, services and interventions” [53]
No definition, but wording suggests that health technologies are products and devices	“Purchasing health technologies” [94–96], “sunk costs and capital infrastructure” [50], “manufacturers” [23, 38, 94, 96], “technology lifecycle” [23, 38, 50], “after a technology has been licensed” [23, 96]

#### Discrepancies in use

The first definition is used primarily in two settings where an all-encompassing description is very useful: by researchers, particularly those working in Health Technology Assessment (HTA), and by policy-makers determining health service coverage. However this broad definition does not reflect common use of the term by health service managers, clinicians or consumers who differentiate between health technologies, clinical practices and healthcare services and programs. Use at local level is better captured by the other three alternatives.

#### SHARE

The SHARE Program used the term ‘technologies and clinical practices’ (TCPs); defined as therapeutic interventions (including prostheses, implantable devices, vaccines, pharmaceuticals and medical, surgical or other clinical procedures) and diagnostic procedures [11, 92]. Health services and programs were referred to separately and not included in the concept of TCPs.

#### Implications for policy, practice and research in the local healthcare setting

It is understandable that some groups need to consider the whole range of health system activities in their work, and obvious that the HTA process and health policy decisions can be applied to “*any intervention that may be used to promote health, to prevent, diagnose or treat disease or for rehabilitation or long-term care including pharmaceuticals, devices, procedures and organizational systems used in health care*” [86]. But by using this catchall as a definition for ‘health technologies’, researchers and policy-makers create potential for confusion and misunderstanding in their communication with health service staff and consumers who use a much narrower interpretation of this term focused on medical products and devices. This may also hamper translation of knowledge about health technologies from research to practice. A definition that captures use at the local level might be ‘products, devices and equipment used to deliver health care (eg prostheses, implantable devices, vaccines, pharmaceuticals, surgical instruments, telehealth, interactive IT and diagnostic tools).’ When this definition is combined with ‘clinical practices’, the term ‘technologies and clinical practices’ reflects the scope of most decisions regarding resource allocation for investment and disinvestment related to health care delivery in the local setting. This terminology will be used throughout this review.

### 1.2 Disinvestment

#### Definition

After more than a decade of research in disinvestment there is still a lack of common terminology [36, 47, 49, 53, 54, 64, 98, 99]. Although the word ‘disinvestment’ occurs most frequently, and has been adopted by several countries in their national programs, multiple terms are used (Table 3). Some terms are used interchangeably with disinvestment [27], new terms have been introduced to capture specific aspects of disinvestment [29, 39], and others proposed to reflect the process of disinvestment more accurately [6].

**Table 3:** Examples of alternatives for the term ‘disinvestment’

Scope	Alternative terms
Used interchangeably with disinvestment	Decommissioning, removing ineffective services, resource release, defunding, rationing [27]
Introduced to capture an aspect of disinvestment	Health technology reassessment [39], de-implementation [29]
Proposed to capture the process of disinvestment better	Displacement, reallocation, reinvestment [6]
Used to avoid the word disinvestment	Prioritisation, reappraisal, reprioritisation, optimisation, substitutional reinvestment, evidence-based reassessment [38], value for money, therapeutic equivalence, allocative reinvestment, reducing waste, bending the cost curve, contract variation, contract management, service redesign [101]

The term ‘disinvestment’ is also used with multiple meanings based on a range of perspectives (Table 4) [27, 64]. Some consider the objective of disinvestment to be reallocation or reinvestment of resources from one TCP to another, while others define it as removal or restriction of use without reference to reallocation. Some definitions are based on the absolute value of a TCP, whether it has intrinsic worth, for example ‘this procedure is not worth funding’. Others compare the relative value of one TCP over an alternative such as ‘practice A has less value than practice B’ where the TCP being disinvested may have intrinsic value but an alternative is thought to have greater value. Some focus solely on TCPs with little or no health gain and others consider a broad range of factors.

**Table 4:** Examples of definitions for ‘disinvestment’

Definition	Measure	Decision criteria	Position	Action
Disinvestment is an explicit process of taking resources from one service in order to use them for other purposes that are believed to be of better value [28]	Any	Less value than available alternative	Relative	Reallocation
Disinvesting in health interventions that offer no or low health gain (eg are unproven, outdated or cost ineffective) provides an opportunity to invest in alternative proven and cost effective health interventions [132]	Effectiveness, Currency, Cost-effectiveness	Unproven, outdated or cost-ineffective	Absolute	Reallocation
Disinvestment is the process of reducing or ceasing health technologies and clinical practices that provide less favourable outcomes than known alternatives [27]	Any	Less favorable outcome than available alternative	Relative	Removal or Restriction
Disinvestment relates to the withdrawing (partially or completely) of health care practices, procedures, technologies and pharmaceuticals that are deemed to deliver no or low health gain and are thus not efficient or appropriate health resources allocations [91]	Effectiveness	No or low health gain	Absolute	Removal or Restriction
Disinvestment can take a number of forms in a healthcare setting…and includes full withdrawal or decommissioning, retraction, restriction and substitution [101]	Any	Unspecified	Unspecified	Removal, Restriction or Replacement
Disinvestment refers to processes by which a health system or service removes technologies, without necessarily replacing them [42]	Any	Unspecified	Unspecified	Removal
Disinvestment relates to the withdrawal of funding from a provider organisation and the subsequent stopping of the service [104]	Any	Unspecified	Unspecified	Defunding (resulting in Removal)
Disinvestment includes the withdrawal or reduction of relatively ineffective healthcare, as well as full withdrawal or rationing of equally worthy alternatives due to resource constraints [60]	▪ Effectiveness ▪ Affordability	▪ Relatively ineffective ▪ Unspecified	▪ Relative ▪ Absolute	Removal or Restriction
Disinvestment: the displacement of non–cost-effective technologies for resource reinvestment or reallocation [118]	Cost-effectiveness	Non–cost-effective	Absolute	Reallocation
Disinvestment involves the development and application of epidemiological, economic, ethical and policy appraisals of existing health care interventions that are cost-ineffective or inappropriately applied within health care, leading to displacement of these practices to make way for resource re-allocation towards practices and programs offering greater benefit [163]	▪ Cost-effectiveness ▪ Appropriate use	▪ Cost-ineffective ▪ Inappropriate use	Absolute	Removal and Reallocation

Many authors cite the definition by Elshaug and colleagues that disinvestment “*relates to the processes of (partially or completely) withdrawing health resources from any existing health care practices, procedures, technologies, or pharmaceuticals that are deemed to deliver little or no health gain for their cost and thus are not efficient health resource allocation*” [91]. Although frequently used, this definition differs considerably with others, particularly those that consider the relative value of TCPs and their alternatives, reallocation of resources released, or financial constraint as the driver of disinvestment decisions.

This mixture of terminology and concepts creates confusion, inconsistency and ambiguity. For example, the term ‘rationing’ is frequently used interchangeably with ‘disinvestment’, and even to define it [60, 64], however the concept of ‘rationing’ does not apply when disinvestment is undertaken to remove a harmful or ineffective TCP [100].

#### Discrepancies in use

There is a discrepancy in use of the term ‘disinvestment’ between the practice setting and the research community. “*Invest to save*”, defined as “*the process of making an investment in the short-term which will bring about savings in the longer-term*”, was identified as the commonest form of disinvestment in one study of health service staff [101], and health service commissioners defined disinvestment as *“limiting new service provision”* in another [64]. Neither of these would be considered to be disinvestment using any of the common research definitions. This divergence is also evident in the lack of definition for disinvestment in many health service publications. The term is used in the context of policies or processes related to “*investment and disinvestment*” with no further explanation of either term [102, 103].

The disparity is not limited to different contexts. In two recent publications, both set in the UK National Health Service, one uses the term ‘decommissioning’ to define ‘disinvestment’ while the other uses a different definition for each word [101, 104]. Inconsistencies have even been identified within the same decision-making body [98].

Further disparity exists in scope of application. Some authors refer to disinvestment of health technologies in the narrow sense of products and devices, some to TCPs, and others note that the concept has been extended beyond individual TCPs to include “*trading-off expenditures between different service groups, better integration of health services between primary and secondary care providers, and better integration of the health system with other government agencies*” [40, 47, 105].

Conflicting terminology also extends beyond the meaning of the term to the process of disinvestment. Some authors stipulate that disinvestment is an explicit process [28, 60, 98] but others consider it to be both implicit and explicit [40]. Although most definitions imply that it is an active process, it has also been classified as active and passive [47, 55, 64]. The same description is used for both explicit and active disinvestment and refers to removal or redirection of funding to achieve practice change. Although the implicit approach is described as passive, it is defined as using education and information dissemination to drive change [40], whereas the term passive disinvestment is used to describe processes that are not reliant on direct intervention by reimbursement policy makers [55] or procedures or treatments that gradually fall out of use over time [26, 47]. While implicit disinvestment potentially leads to more co-operative and flexible means of identifying areas for disinvestment; it may be ineffectual and may be more difficult to attribute savings or improvement in patient outcomes to disinvestment. The explicit approach potentially captures savings more convincingly; but the risk is loss of stakeholder support [40, 56].

#### Negative connotations

In the absence of common terminology, there is one notably consistent message: that the word ‘disinvestment’ has negative connotations and is likely to be a barrier to successful implementation of disinvestment-related change. It is associated with ‘taking away’, has a perceived focus on cost cutting, is associated with ‘top down’ interference and implies a criticism of current practice [27, 38, 46, 49, 50, 64, 98, 106]. To reduce undesirable effects, other terms have been intentionally introduced to replace ‘disinvestment’ (Table 3) [38, 101].

#### Theories, frameworks and models

Theories, frameworks and models for disinvestment are discussed more fully in Paper 10 of this series [84]. A summary is presented here in consideration of terminology and concepts related to disinvestment.

There is little discussion of the role of theory or theoretical approaches to the concept of disinvestment [84]; however the theory of discontinuance, part of the theory of diffusion of innovations [107], has potential for disinvestment in health care [98, 108]. While no theories of the overall process of disinvestment were identified, several theories have been applied in projects investigating decision-making in this context [45, 53, 109–112].

Fifteen frameworks and models related to disinvestment, resource allocation and priority setting were identified [84]; however they are mostly conceptual and as yet untested. They address projects to identify and disinvest individual TCPs [53, 113–116], programs for sector-wide investment and disinvestment [103, 106, 117, 118], evaluation [63, 114, 119] and stakeholder engagement [103, 120].

#### SHARE

The definition of disinvestment used in early development of the SHARE Program was “*cessation or limitation of potentially harmful, clinically ineffective or cost-inefficient TCPs*”, which takes the absolute position. This was later expanded to include the relative position for the pilot disinvestment projects which were defined as activities that *“remove a TCP that is unsafe or ineffective, restrict a TCP to more appropriate patient groups, or replace a TCP with an equally safe and effective but more cost-effective option”.*

Although the SHARE Program made a decision to avoid the term disinvestment, a suitable alternative proved elusive for one of the main program components which was known throughout as the “*Disinvestment pilot projects*” [114].

Several frameworks and models were developed in the SHARE Program; these are presented in detail in the relevant papers and are summarised in Paper 10 [84]. The frameworks include potential settings and methods to integrate disinvestment decisions into health service systems and processes [113], components in the resource allocation process [117] and evaluation and explication of a disinvestment project [114]. The models include integrating consumer values and preferences into decision-making for resource allocation in a local healthcare setting [120], exploring Sustainability in Health care by Allocating Resources Effectively in this context [106] and facilitating use of recently published synthesised evidence in organisational decision-making through an Evidence Dissemination Service [115]. An algorithm facilitates decision-making for developing a disinvestment project from an evidence-based catalogue of potential opportunities for disinvestment [114]. A framework for evaluation and research was also developed for the whole SHARE Program [121]. A framework for organisation-wide disinvestment in the context of resource allocation is proposed in Paper 10 [84].

#### Implications for policy, practice and research in the local healthcare setting

A common understanding of terminology and concepts is required for successful decision-making, communication and implementation of change in the policy and practice settings. A consistent definition is also important for evaluation of change in the practice setting and activities in the research domain to increase rigour, ensure validity of outcomes, enable replication and comparison with others, facilitate application in equivalent situations to reduce duplication, engender familiarity and understanding to increase uptake and use of content, and build on existing work. The current multiplicity and variability of definitions hampers these objectives.

In the absence of common terminology, a definition and the concepts underpinning it should be established for shared understanding by stakeholders of disinvestment initiatives. However, the literature recommends that the term disinvestment should be avoided when attempting to implement change. A different word or way of capturing and framing these concepts to facilitate the related activities may be preferable.

Another approach could be to simplify the definition of disinvestment to ‘removal, reduction or restriction of any aspect of the health system’. Removal indicates complete cessation, reduction is a decrease in current volume or delivery sites, and restriction is narrowing of indications or eligible populations. This could apply equally to devices and equipment, clinical practices and procedures, health services and programs. In the same way that investment is a process of allocating resources for the introduction, continuation or expansion of any aspect of the health system, disinvestment could simply be the decision to remove, reduce or restrict and not be complicated by the type of activity undertaken. An understanding of how the word disinvestment is being used in a particular setting would no longer be necessary and use of the word as the basis for an activity would become redundant. The focus could then be the valid reason for change, such as patient safety or reducing waste, and not the negative perceptions of the word or the notion of disinvestment for the sake of disinvestment.

Unless otherwise specified, disinvestment is considered in its broadest sense, ie according to the definition above, throughout this review.

### 1.3 Resource allocation

Disinvestment is frequently presented as an isolated activity independent of other decision-making processes, to be pursued for its own ends. Investment as a concept is rarely noted in the disinvestment literature. Yet in practice, investment and disinvestment exist together at opposite ends of a continuum [39, 50, 106]. When a new TCP is found to have greater benefit than an existing one, it implies that as one is introduced the other should be removed, either partially or completely. Introduction of a new TCP provides a trigger to explore opportunities for disinvestment [26]. Investment without appropriate disinvestment can be wasteful and making disinvestment decisions outside the context of existing decision-making processes may result in unsuitable or unsustainable outcomes [106]. Decisions about investment and disinvestment can be considered together as ‘resource allocation’ [117, 122].

Discussion about investment, disinvestment and reinvestment in the literature is usually focused on decisions about money, yet many decisions in healthcare, particularly at the local level, are about use of non-monetary resources and are often driven by considerations other than financial constraint [113]. Resource allocation is an inclusive term that encompasses financial and other resources. It also draws the focus away from the cost of healthcare provision and the perception that decisions to remove or reduce things are always about money and redirects it towards the idea that resources are limited and should be targeted to achieve the best outcomes [106].

Many national and regional policies are now based on resource allocation and address both investment and disinvestment [102, 103].

#### SHARE

Resource allocation is embodied in the name of the SHARE Program: Sustainability in Health care by Allocating Resources Effectively. It was made explicit that the program covered the spectrum of decision-making from investment to disinvestment and included monetary and non-monetary resources.

#### Implications for policy, practice and research in the local healthcare setting

Investment decisions usually have inherent incentives for successful implementation as they enable continued availability of practices in regular use or facilitate introduction of improvements to current practice. Conversely, if disinvestment activities are not considered in the context of other decision-making processes, they introduce inherent disincentives through loss of things that were familiar and believed to be beneficial without the balance of positive alternative outcomes. If the frame of reference is ‘resource allocation for maximum effectiveness and efficiency’, with the focus on enhancing patient outcomes and using limited resources wisely, the reasons for disinvestment and the resulting benefits become evident and provide some incentives for change.

### 1.4 Optimisation of health care

Sometimes the considerations for change are not as straightforward as ‘to fund or not to fund’ or ‘x is better than y’ [45, 93]. In addition to unsafe, ineffective and inefficient TCPs, many authors propose that inappropriate use of therapeutic interventions, systematic errors and organisational waste should also be addressed, and that a wider consideration of ‘optimising health care’ is preferable to disinvestment alone [23, 34, 38, 39, 50, 85, 123].

TCPs with demonstrated safety and effectiveness may still pose a problem if used inappropriately. Overuse, underuse or misuse may be inadvertent due to lack of knowledge or skill [23, 24, 48] or intentional due to a range of other factors [62, 124]. There may be isolated errors, but if the problem is widespread due to systemic issues such as entrenched practices, poor training or inadequate staffing it will result in significant waste of resources. In these situations the target for disinvestment is the inappropriate use of a TCP rather than the TCP itself. The term ‘disinvestment’ is not widely used in the American healthcare context, however the national ‘Choosing Wisely’ and ‘High Value Care’ initiatives to improve health outcomes and reduce costs are focused on decreasing waste and reducing inappropriate use of therapeutic interventions [68, 125, 126]. This approach is being replicated in national campaigns around the world [127].

Another reason to consider the optimisation perspective is that it may circumvent the ethical dilemmas associated with other approaches to disinvestment. Clinicians are required to follow the principle of beneficence, to act solely in their patient’s best interests and to advocate on their behalf; however this conflicts with the principles of justice and fairness that necessitate rationing of finite resources [31, 68, 100]. Similarly there may be conflict between the principles of equity and efficiency in cases where the most efficient program identified by a disinvestment process is not the most equitable [105, 128, 129]. ‘Return on investment’ is a concept being introduced into the disinvestment debate, however ethical conflicts between return on investment and the principle of preventing ill health and the human right to health have been acknowledged [105]. Reducing inappropriate care and eliminating waste is compatible with beneficence, equity and efficiency, prevention of ill health and the basic human right to health and consistent with the disinvestment aims of removing harmful or ‘low value’ practices.

An optimisation approach has also been proposed to address the difficulties related to finding the unequivocal evidence of harm or lack of effect required for disinvestment decisions. ‘Optimal targeting’ has emerged as an alternative strategy where the focus is on identifying the subgroups for which a TCP is most clinically or cost-effective [1, 10, 38, 55, 56, 59]. Rather than disinvestment, this is referred to as “*refining the indications for service provision*”, targeting TCPs to those who will benefit rather than removing them from those who will not [45].

#### SHARE

‘Optimising health outcomes’ was not an overt principle in the SHARE Program where the focus was stated as ‘effective application of health resources’. However it was implicit in all the activities and often explicit in presentations and explanations of the approach. One of the key components of the program was investigation of decision-making processes to identify systematic problems and opportunities for improvement [117] and another was exploration of potential disinvestment projects, several of which were based on inappropriate use [114].

#### Implications for policy, practice and research in the local healthcare setting

Improving health outcomes is a fundamental objective of health care and a primary motivator for healthcare staff. Initiatives that emphasise the positive approach embodied in allocating resources to optimise health care may be more welcome than those focused on disinvestment with its inherent negativism.

Inappropriate use of TCPs, systematic errors and practices resulting in organisational waste should be removed because they harm patients, diminish health outcomes, impair health care delivery and increase costs unnecessarily. If opportunities for disinvestment are being sought, it could be argued that these issues are addressed first, before considering removal, reduction or restriction of procedures or processes that have relatively less benefit than available alternatives but which have intrinsic value of their own [115].

### 1.5 Reinvestment

The terms ‘reinvestment’ and ‘reallocation’ appear to be used with the same or similar meaning in the literature; however, like investment, they are not defined. They are variously considered to be the objective of a disinvestment exercise [28, 53, 130], the expected result [38, 39, 122], a ‘hoped for’ outcome [47, 61, 85, 131, 132] or not mentioned at all.

There is an assortment of views on the proposed targets or beneficiaries of reinvestment. Some specify that resources freed up through disinvestment of ‘low value’ TCPs should be redirected to TCPs that deliver safe and effective healthcare [37, 38, 116]. Another perspective is for resources to be retained by the group undertaking the disinvestment activity or to be used for the benefit of patients with the same condition or to improve care in the same specialty area [50]. In contrast, some make the case that there should be no expectation that resources are returned to the same area and that it may be most appropriate to reinvest in another service or TCP [40, 53, 122]. Others note that the purpose of disinvestment can range from identifying resources specifically for reallocation or reinvestment through to finding savings to meet budgetary shortfalls where the intention is not to reinvest or reallocate but to put the released funds towards “*the bottom line”* [101, 133].

Resources theoretically released through disinvestment may not be achieved in practice. For example, reducing length of hospital stay may be anticipated as a saving of ‘bed days’ but, unless the beds are actually closed, they will be occupied immediately by a different patient group [117, 134]. This is a positive outcome as it gets some patients home earlier and reduces waiting times for others, but it is not a saving. There is also potential for disinvestment in one area to increase costs or resource utilisation in another; a practice change may avoid the need for surgery but the patients require additional outpatient services [85, 117]. And it is possible that the costs of developing, implementing and evaluating a disinvestment initiative will be more than the expected savings [135].

No formal methods for quantifying savings and benefits from disinvestment or implementing a reinvestment plan have been proposed and this deficiency has been noted as a significant barrier [51, 60, 123, 136].

#### SHARE

It was acknowledged early in the SHARE Program that reinvestment would not be possible as local accounting methods and the inability to itemise expenses for complex activities spanning multiple budgets and cost centres precluded measurement of savings from disinvestment projects.

#### Implications for policy, practice and research in the local healthcare setting

For reinvestment to occur resources must be released, be measured and be made available for reallocation. Any or all of these may not be achievable.

## 2. Motivation and purpose

Definitions and terminology related to disinvestment are debated in the literature, however there is little consideration of the impetus and rationale for undertaking disinvestment [57]. The reasons underpinning specific disinvestment activities are likely to affect all aspects of the process from identification and prioritisation through to implementation and evaluation but this has not been widely discussed.

### 2.1 Impetus for disinvestment

The drivers for disinvestment have varied over time and within and between settings. An example of this is the change in approach to disinvestment by the UK National Health Service. In 2002 a “*need to maximise efficiency and abandon ineffective interventions*” was recognised; in 2005 the concept of “*value for money*” was added; in 2006 this was quantified in a pilot project “*to identify individual low value interventions which if stopped would save over £1m each*”; and in 2011 external financial pressures introduced “*cost saving*” as a primary driver of disinvestment [10]. These are four different objectives that will require different approaches to identification of disinvestment targets, decision-making, implementation and evaluation and have potentially different timeframes and resource requirements.

There is also a difference between rhetoric and practice. A recent international study found that disinvestment experts thought that the main drivers for disinvestment should be safety, effectiveness and cost-effectiveness, but in their experience budgetary pressures, government intervention, and capital costs and conditions were the actual reasons for change [57].

Drivers for disinvestment at the national level are likely to be based on evidence of harm, lack of effect, or availability of a more cost-effective alternative, where the evidence can be applied broadly. But local factors might identify disinvestment opportunities that are not generalisable to all health services. A study surveying local commissioners of health services across England concluded that the context for decision-making is more important than the deployment of specific tools and techniques and, in the absence of a formal process, the choice of approach would be influenced by the objectives of individual initiatives [105].

### 2.2 Rationale for disinvestment

It has been noted that the reasons for undertaking disinvestment can vary [101] and that project objectives are not always clear in research publications [26]. The reported aims have also been described as intertwined and unable to be delineated [56]. Disinvestment has been described as addressing three health system imperatives: ethical, quality and economic [76] but no other descriptions or classifications of the reasons for disinvestment were identified.

**Fig. 1:**
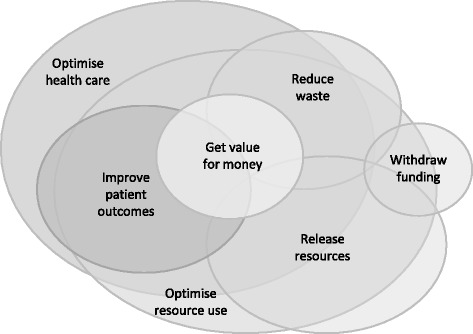
Relationships between reasons for disinvestment

Many of the multiple definitions include or imply a reason for disinvestment. This wide range of concepts can be summarised in seven main themes (Table 5). An eighth option, ‘for any reason’, is added for completeness. Some of these concepts are broad and others quite narrow. There is considerable overlap between some themes, for example ‘improving patient outcomes’ and ‘getting value for money’ could both be objectives shared by projects focused on ‘optimising health care’ (Fig. 1). However others might be mutually exclusive. A project to ‘improve patient outcomes’ based on replacing an ineffective treatment with an effective, but more costly, alternative is not compatible with another aiming to ‘release resources’ or ‘withdraw funding’.

**Table 5:** Examples of reasons for disinvestment from the literature

Objective	Scope
Any reason	This is the broadest sense of disinvestment and refers to cessation or limitation of something that was previously in practice. It could apply to services, programs, use of equipment, diagnostic tests or therapeutic interventions. Words used interchangeably with disinvestment in this context are decommissioning, de-implementation, removal, replacement, restriction
To optimise health care	This is also a broad concept. It incorporates investment, disinvestment and reinvestment. The focus is on effective allocation of resources to achieve maximum benefit and combines the concepts of safety, effectiveness, cost-effectiveness and eliminating waste. The approach of ‘optimal targeting’ is also captured here.
To optimise resource use	A similarly broad concept to optimising health care with considerable overlap of intentions. The difference is in the emphasis on economic outcomes rather than other aspects of health care. This is the objective of Program Budgeting Marginal Analysis (PBMA) and other prioritisation activities.
To improve patient outcomes	This relates to removal of harmful or ineffective practices which result in adverse outcomes for patients and/or replacement with more effective alternatives. The focus is safety and effectiveness but the terms ‘low value’ and ‘of little or no health gain‘are also used in this context. There is potential to increase costs rather than save money.
To reduce waste	This could also be thought of as improvement in health service outcomes. From the perspective of disinvestment this primarily addresses inappropriate use of diagnostic tests and therapeutic interventions and failure of care coordination.
To get value for money	This is based on consideration of cost-effectiveness and/or risk-benefit analysis. It may be defined by specifying acceptable cost/QALY ratios or based on local values.
To release resources	This can have two elements: to save money in times of financial constraint or to redirect funds to a preferred alternative. Terms used in this context are cost saving, rationing, priority setting, reinvestment and reallocation. Priority setting exercises may also have this as an objective to use disinvestment to enable investment.
To withdraw funding	The focus of this concept is on the process of disinvestment rather than the reason for doing it. Disinvestment defined in this way refers to the act of withdrawing funding from a provider organisation which results in cessation of a service.

There are many more reasons for undertaking disinvestment than those captured in the seven themes noted from the literature, particularly from the perspective of a local healthcare service. A list of potential reasons for individual disinvestment projects is presented in Table 6. This is illustrative rather than exhaustive and the utility of the categorisation is untested. Some items are very specifically aiming to disinvest, such as discontinuing a service in order to save money, but the majority are examples of aims to address common problems in the health system where disinvestment is a possible solution. Some of these may overlap with others and some are very similar with only subtle differences in context or emphasis. This list is submitted to prompt debate and further research exploring whether making the rationale for disinvestment explicit is a barrier, enabler or determinant of successful disinvestment and what difference the variations in context and emphasis may have.

**Table 6:** Potential reasons for disinvestment in the local healthcare setting

External ▪ To address political priorities ▪ To meet legislative, regulatory or accreditation requirements and professional standards ▪ To meet national recommendations ▪ To address legal and ethical issues ▪ To be sensitive to the environment
Financial ▪ To save money to meet budget cuts ▪ To find money to spend on something else ▪ To prioritise where money is spent ▪ To redistribute within or between budgets ▪ To support investment in new technologies ▪ To support continued investment ▪ To get value for money
Economic ▪ To maximise benefits from resource use ▪ To improve efficiency ▪ To maintain quality without extra expenditure ▪ To remove TCPs with unacceptable cost per QALY
Organisational ▪ To meet strategic goals and priorities ▪ To ensure sustainability ▪ To increase productivity ▪ To work within organisational capacity ▪ To work within staff capability ▪ To rationalise services eg only provide orthopaedics at hospital A and oncology at hospital B ▪ To enable system redesign ▪ To reduce health service utilisation ▪ To reduce risk to staff, finances or reputation ▪ To reduce waste ▪ To address specific problems
Patient care ▪ To improve patient health outcomes ▪ To reduce patient harm ▪ To target populations or indications for best results ▪ To improve patient flow and reduce waiting times ▪ To improve patient satisfaction or reduce inconvenience ▪ To improve patient access and equity of service provision ▪ To reduce unnecessary tests or treatment
Health technology, clinical practice or service ▪ To keep equipment up-to-date ▪ To remove obsolete or superseded technology ▪ To remove or restrict TCPs that are harmful ▪ To remove or restrict TCPs that have little or no value ▪ To replace TCPs with alternatives of greater benefit ▪ To remove services that are not performing as intended ▪ To remove services that are not meeting the needs of the target population
Evidence Based Practice ▪ To ensure practice is consistent with current evidence ▪ To actively identify evidence of harm or lack of effect and remove relevant TCPs ▪ To update evidence-based guidelines and protocols
Social judgement ▪ To ensure public funds are spent wisely ▪ To reduce public funding on discretionary services eg some cosmetic procedures

#### SHARE

The SHARE Program used two main approaches. A broad approach was taken with the pilot projects, TCPs could be proposed for disinvestment for any reason [114]. However use of an Evidence Dissemination Service to identify potential disinvestment targets from recently published high quality research was more specific, focusing on evidence of harm or availability of more cost-effective alternatives [115].

#### Implications for policy, practice and research in the local healthcare setting

The range and diversity of reasons for disinvestment, and the complexity of relationships between them, add to the difficulties in considering disinvestment as a single entity in anything other than the broadest sense. The comprehensive simplified definition for disinvestment proposed in the preceding section could be extended to ‘removal, reduction or restriction of any aspect of the health system *for any reason*’, in the same way that investment is a process of allocating resources for the introduction, continuation or expansion of any aspect of the health system for any reason. The motivation and purpose in individual situations can then be used explicitly in development of project objectives and strategies without the limitations and complications of being embedded in a definition.

Consideration of the reasons for disinvestment is crucial to project planning. If the objective of a disinvestment activity is specifically to reinvest, the savings need to be measured and explicit decisions about redeployment of the funds are required. However if the purpose is to reduce patient harm or improve health outcomes, the evaluation parameters will be patient measures and there may no savings to reinvest and possibly increased costs to find. The barriers and enablers to implementation and evaluation of these two scenarios are likely to be quite different.

## 3. Relationships with other healthcare improvement paradigms

A paradigm is a framework containing the basic assumptions, ways of thinking, and methodologies that are commonly accepted by members of a scientific community [137]. Disinvestment is frequently presented as if it is a new paradigm for health improvement. It has been described as an ‘emerging field’; disinvestment approaches, processes and initiatives are discussed; ‘research agendas’ are considered; and the need for mechanisms, frameworks, methods and tools are widely acknowledged [26, 35–39, 47, 49–51, 56, 88, 90, 91, 98, 101, 105, 123]. However there are several health improvement paradigms with mature frameworks and validated methods and tools that are widely-used and well-accepted in local health services that already undertake disinvestment-type activities.

### 3.1 Evidence-based health care

Disinvestment is intrinsically linked to evidence-based health care (EBHC). A fundamental tenet of EBHC is that practices found to be harmful, ineffective or inefficient should be removed and an evidence-based approach would also routinely identify alternatives that were more effective or cost-effective than existing practices. Disinvestment is a natural outcome of EBHC.

While it would be possible to disinvest without taking an evidence-based approach, there is a strong consistent theme in the disinvestment literature advocating for explicit use of evidence in decision-making [6, 23, 26, 37–39, 42, 50–53, 58–61, 88, 95, 100, 104, 111, 131, 138–140]. The triad of evidence, expertise and consumer perspectives which underpins EBHC is also common to most publications on disinvestment.

Systematic reviews are the foundation of EBHC and are often represented in the disinvestment context as Health Technology Assessments (HTAs) or Health Technology Reassessments (HTRs), a term coined more recently to specify appraisal of existing, rather than new, TCPs with view to identifying potential targets for disinvestment [39, 85]. There are numerous examples in the disinvestment literature promoting this evidence-based approach and exploring methods to initiate and undertake HTA/HTR and implement the findings [6, 12–14, 23, 26, 39, 41, 50, 51, 61, 95, 131, 141]. Proactive use of Cochrane systematic reviews has been employed to create national recommendations for disinvestment [10]. Evidence-based guidelines have been proposed as vehicles for implementing disinvestment decisions [10, 28, 42, 46, 139].

Disinvestment is also entwined with three new fields of research and practice that have emerged from the EBHC movement: Comparative Effectiveness Research, Knowledge Translation and Implementation Science. Finding existing evidence, generating new evidence to fill gaps, appraising and synthesising it, getting it to decision-makers, using it in decisions and implementing the appropriate changes are all highlighted in the disinvestment literature.

#### SHARE

The SHARE Program was explicit in taking an evidence-based approach [106]. The SEAchange model for Sustainable, Effective and Appropriate evidence-based change was used for development, implementation and evaluation of the program components and projects [142]. Methods to use evidence from research and local data proactively to drive disinvestment decisions were explored [115, 143].

#### Implications for policy, practice and research in the local healthcare setting

Cessation or limitation of practices occurs regularly as a result of evidence-based processes. In the EBHC context this can be achieved in a positive sense by ‘implementing best practice’ and the negative term ‘disinvestment’ can be avoided.

There are two significant bodies of literature in disinvestment that can be distinguished by their approach to evidence and the sources they use: those focused on use of HTAs are driven by evidence from the research literature on the safety, effectiveness and cost-effectiveness of TCPs and those focused on priority setting where decisions are based on evidence from stakeholders, local health service utilisation data and economic factors. Used separately these sources of information are insufficient for robust decision-making at the local health service level; they are both required [113]. Evidence from research can highlight potential targets for disinvestment but before changes are proposed this information should be considered in light of local data. If an issue only affects a few patients or practitioners, or the burden of disease and hence potential impact are small, particularly in comparison with other issues, resources for change may be better employed elsewhere. Similarly, evidence from local data can identify problems, however review of known best practice from the published literature is required to identify effective potential solutions [113].

Most of the research in EBHC has been conducted in the domain of clinical practice. While there is still much to learn, there is a substantial evidence base to guide knowledge translation strategies for health professionals and consumers. However the main focus of disinvestment has been on policy and management decisions where the evidence for knowledge translation is much weaker [144–146]; identifying potential for future research.

### 3.2 Quality improvement

For many authors writing in the area of disinvestment, quality and cost are integrally related in their arguments; even noted as “*two sides of the same coin*” highlighting the tension created by the expectation that health services will deliver better care while reducing costs [147]. Savings and improved quality of care have been cited as the two main objectives of disinvestment [38, 48, 132]. From a big picture perspective, disinvestment can be seen as part of a broader policy agenda to improve efficiency and quality of care [10, 45]; and from a local perspective, disinvestment is seen to deliver quality care as it is embodied by the definition ‘the right care at the right time in the right place’ [10, 70, 101].

Disinvestment policies are frequently linked to quality improvement (QI) instruments such as plans, programs or institutions. Examples of national disinvestment policies linked to quality vehicles include the Spanish National Health System Quality Plan [37], Australian Medicare Benefits Schedule Quality Framework [43], UK Quality, Productivity and Prevention Programme [10], Norwegian Council for Quality Improvement and Priority Setting in Health Care [39], and the German Institute for Quality and Efficiency in Health Care [42].

The benefits of the formal linkages between disinvestment and QI could potentially flow in either direction or be mutually beneficial. Disinvestment might be a useful tool to achieve QI objectives. It has been described as “*a driver, and an enabler, of patient safety and quality health care provision*” [38]. Some authors anticipate that disinvestment can reduce costs without reducing quality [101, 130], but many more expect that disinvestment will result in improved quality [28, 34, 39, 40, 45, 50, 85, 91, 116], and others describe both outcomes [10, 38]. Alternatively, linking disinvestment with quality initiatives could increase the likelihood of successful implementation [38, 85]. This is thought to facilitate more transparent discussions [45], redirect negative perceptions of rationing or cost cutting towards the positive objectives of quality and safety [46], focus on standards and performance indicators [23], and make it more acceptable to clinicians and consumers [38, 50].

Many examples of disinvestment are described in the quality improvement literature. For example interventions to decrease adverse events; limit overuse, underuse and misuse of treatments; and reduce duplication in service delivery are all thought to save money [135] and would meet any of the definitions of disinvestment, yet are most frequently referred to as ‘quality improvement initiatives’ and the term ‘disinvestment’ is never considered. There are also many examples of harmful practices being ‘disinvested’ but the literature describes these as ‘patient safety strategies’ [148, 149].

#### SHARE

The SHARE Program linked to the Quality Program at Monash Health through the Policy and Procedure Framework. A new framework was developed by the SHARE team and implemented by the Quality Unit. Guidance for developing new and revising existing policies and procedures included steps to identify potential TCPs for disinvestment [114].

#### Implications for policy, practice and research in the local healthcare setting

Quality improvement is a much wider and more mature field of policy, practice and research than disinvestment, but given the parallels in objectives, it might prove to be a valuable source of information about methods for decision-making, implementation and evaluation.

### 3.3 System redesign

System redesign involves systematic changes to organisational processes to improve health outcomes, enhance patient and staff experiences of care, and increase efficiency [114]. It uses an array of approaches rather than a single technique, and has significant overlaps with EBHC and QI.

‘System redesign’ has been used synonymously with ‘disinvestment’ and proposed as a method to identify disinvestment opportunities, implement disinvestment decisions and/or quantify disinvestment outcomes [38, 60, 101]. Invoking the term ‘system redesign’ has also been suggested as a strategy to increase the likelihood of implementation by avoiding the word ‘disinvestment’ [101, 136].

#### SHARE

System redesign was investigated through a literature review and interviews with Monash Health staff experienced in this area. A decision was made that these processes would be considered as implementation strategies for the pilot disinvestment projects [114].

#### Implications for policy, practice and research in the local healthcare setting

Like EBHC and QI, system redesign is familiar to health service staff and offers a well-established and accepted context to introduce practice change [113]. The methods used can identify disinvestment opportunities, implement the decisions and evaluate the outcomes.

### 3.4 Health economic approaches

Most of the early research in disinvestment was based on health economic principles, primarily priority-setting approaches. Historically, priority-setting was an exercise to decide between investment options, however the current economic challenges in health care have led decision-makers to consider disinvestment strategies in this process [60].

There are many priority-setting approaches [150], the most common being Program Budgeting and Marginal Analysis (PBMA) [133] which now features highly in the literature as a rigorous, transparent method to identify disinvestment opportunities. PBMA applies the economic principles of opportunity costs and margins to determine priorities for health program budgets in the context of limited resources [151]. The language of the PBMA framework has changed over the past decade to make disinvestment more explicit. In 2001 the framework sought to release resources through increasing effectiveness and efficiency [152]; in 2004 it noted “*scaling back or stopping some services*” as one way to release resources [153] and by 2010 “*evaluation of investments and disinvestments*” had become an overt component [154]. PBMA has been proposed as the basis of a pragmatic framework for “*rational disinvestment”* that can incorporate service redesign approaches [155] and some successes in this context have been reported [156, 157].

#### SHARE

Monash Health did not have a health economist and chose to take an evidence-driven, rather than economic-driven, approach to disinvestment based on the in-house expertise in utilisation of evidence from the research literature and local data [106]. However a consultant health economist was engaged to work with the SHARE team to advise on design and evaluation of program components and projects. The potential for PBMA to be used for disinvestment at Monash Health was explored, but it was decided that without in-house expertise this was not a feasible option [114].

#### Implications for policy, practice and research in the local healthcare setting

Unlike EBHC, QI and system redesign, health economics methods are not familiar to most staff in health services. PBMA and other priority-setting approaches have been employed by university health economists working with health sector decision-makers in research projects. Although they have considerable potential benefits, implementation of these methods in routine decision-making will require academic partnerships and appropriate funds.

## 4. Challenges

In addition to the lack of common terminology, negative connotations of the term ‘disinvestment’, shortage of theories and tested frameworks and models, and paucity of proven methods and tools, the nature of disinvestment brings some particular challenges for policy-makers, managers, health professionals and researchers working in this area.

### 4.1 Sense of loss

The aversion to loss described in prospect theory is particularly relevant to disinvestment [158]. Clinicians and patients perceive greater disadvantage from removal of a TCP, program or service in current use than denial of access to a new one of similar value [50, 99]. Patients also feel entitled to services previously available to them and removal results in loss of that entitlement [50, 55, 134]. The perceived loss from disinvestment is clear and immediate, while any gains from disinvestment may not be readily specified, may not occur for some time, and may not even be achieved at all [42]. For clinicians, removal of a TCP, program or service is not only a loss of something they believed was beneficial for their patients, but also a loss of autonomy [99]. The emotions arising from loss can create formidable opposition that must be anticipated and dealt with [38, 42, 50, 56].

### 4.2 Challenge to clinical expertise

Health practitioners choose tests and treatments based on what they believe to be the patient’s best interests [64]. A decision to remove, reduce or restrict a technology or clinical practice in current use introduces criticism or potential censure of their expertise. It is challenging for clinicians to accept that current evidence may demonstrate that the care they have provided in the past was less than ideal [98, 99]. Clinicians may also see specific practices as integral to their professional practice and identity, making change particularly difficult [50, 55, 91, 159].

### 4.3 Need for more convincing evidence

To overcome stakeholder resistance, the evidence for removal of a TCP, program or service must be more persuasive than for introduction of a new one [38, 42, 50]. Not only is convincing evidence of absence of benefit required, but also evidence of absence of harm from its withdrawal. While more information and less uncertainty are required [10, 50], the reality is that there is a lack of conclusive evidence for most current practices [26, 48, 51, 56, 100]. Finding evidence for existing practice is more difficult than for new practices which routinely have randomised controlled trials to support them [50, 87]. Since current practice is assumed to be of benefit, conducting trials that question this assumption face resistance, potential ethical objections, impediments to funding and difficulties in recruitment.

### 4.4 Possibility of benefit

Potential targets for disinvestment are often identified from evidence of harm or lack of benefit. These research findings are based on outcomes of the total study population or specified subpopulations. However there is always a possibility that the TCP may be of benefit to other subgroups or some individuals [10, 20, 50, 56, 76]. Individual patients who experience improvement from a current treatment and clinicians who perceive benefit in certain patient groups can argue for exceptions. There are also situations of ‘last resort’, when all other treatments have failed or there is imminent risk of death. Flexibility in implementation of disinvestment decisions in these circumstances could be considered [10, 50, 100].

### 4.5 Heterogeneity of outcomes

A diagnostic or therapeutic intervention can have multiple outcomes. It may result in benefit, have no effect, or even cause harm when used in different patient groups. Effectiveness identified in a particular population with certain indications may not be evident in another group with different characteristics [10, 38, 48, 56, 76]. Disinvestment is generally thought of from the perspective of a dichotomous decision: to maintain or to remove. Selective removal from some patient groups or restriction to certain indications is more complex to communicate as a disinvestment decision and becomes a much more difficult task to implement [55]. This complexity increases when the reason for disinvestment is inappropriate use of TCPs in a patient group. The decisions become more controversial when the service or practice is effective, but does not reach a specified cost-effectiveness threshold, or there is another of equal effect which is more cost-effective [48].

### 4.6 Lack of data

There is a universal lack of suitable economic and usage data and no formal methods for quantifying savings and benefits from disinvestment [10, 51, 56, 100, 135]. Current routinely-collected datasets are considered to be generally inadequate, however improving their quality and reliability may still not address the problem. They lack the precision required for disinvestment and the expense of customisation to achieve this is likely to be prohibitive [10]. Data is needed to underpin decisions, support implementation strategies and monitor and evaluate outcomes. Measurement of savings enables reinvestment and provides incentives for future disinvestment. Without appropriate data and the ability to measure resource release, the concept of disinvestment is undermined.

### 4.7 Lack of standardised practices/Lack of transparency

The absence of standardised methods for disinvestment decision-making is well-recognised [51, 57, 101, 123] and lack of transparency is also discussed in relation to disinvestment processes [38, 50, 57, 64, 88, 105, 114, 133]. The ad hoc approaches commonly used, based on *“gut feeling”* and the search *“for a quick fix”* [57], are reported to be *“non-sustainable, reliant on chance or not conducive to independently identifying local opportunities for disinvestment”* [98].

### 4.8 Conflicting roles of local decision-makers

In regional and local healthcare settings, those making decisions to disinvest are likely to have multiple roles [117]. As clinicians they are advocates for their patients; as managers they are advocates for their departments; as decision-makers considering disinvestment they are advocates for the healthcare system, wider population, principles of effectiveness and efficiency, or whatever concepts underpin the local process. There is potential for these roles to be conflicted and it is understandable that the personal, practical and immediate needs of patients and colleagues may be given greater priority than the less tangible and more distant outcomes of disinvestment.

### 4.9 Nomination by ‘outsiders’

There are two issues at play here. Firstly, when invited to nominate candidates for disinvestment, clinicians frequently identify the practices of other professional groups rather than their own [74, 98, 114]. This may induce resistance in those whose practice is being challenged by others outside the relevant area of expertise and preclude local ownership of the problem making successful implementation less likely. Secondly, *“how the technology got on the agenda, where it came from and who was pushing for it”* have been reported as important factors for senior health decision-makers [88]. The influence of nominations from ‘outsiders’ may introduce unnecessary conflict or bias in the decision-making processes.

### 4.10 Lack of clarity and rationale

Clarity of aims and objectives at the start of a project and clear rationale for change were in the top 10 considerations for successful disinvestment, one of three best practice recommendations arising from a study of international experts [57] and one of three key themes from an international workshop [85]. Lack of clarity and rationale has been reported as a problem in identifying suitable disinvestment projects. Insufficient information on the population, intervention, comparators, outcomes, harms and benefits, strength and quality of evidence, and wider implications of the proposed change are noted as the main issues [48, 114].

#### SHARE

All of these were experienced in the SHARE program. Summaries of findings related to these challenges presented in the SHARE papers include: issues to consider in development of an organisational program for disinvestment [113]; implications for disinvestment in the local setting and resulting decisions for program development [106]; barriers and enablers to implementing and evaluating health service decisions for resource allocation [117]; and factors that influenced decisions, processes and outcomes in undertaking disinvestment projects [114] and establishing services to support EBHC [143].

#### Implications for policy, practice and research in the local healthcare setting

Decision-making in healthcare is described at three levels: macro (national, state/provincial and regional settings), meso (institutions) and micro (individuals) [141, 160]. At macro and meso levels, governments and institutions can withdraw funding or issue guidelines, but enacting these recommendations requires change at meso and micro level [70, 139, 161]. In addition, some decisions cannot be made centrally. National recommendations cannot take into account local factors such as population demographics, organisational priorities, budgets, capacity or capability; hence many decisions about the use of TCPs, programs and services have to be made locally [11]. The challenges inherent in disinvestment processes, particularly those related to implementation, are likely to have greatest impact in the local healthcare setting.

## New approach to disinvestment

Although research and debate in disinvestment is increasing, and several countries have formal programs, there is little evidence of active and successful implementation of specific ‘disinvestment initiatives’ in the local healthcare setting [42, 47, 51, 56, 64, 101]. Seeking out targets when the expressed aim is ‘to disinvest’ has not been effective [10, 26, 48, 101, 105, 114]. This review highlights many reasons why this might be so.

However successful removal, reduction and restriction of technologies, clinical practices, programs and services are commonplace at the health service level; but these changes have not been called disinvestment. In these cases, the impetus for change is not ‘to disinvest’ but to meet more constructive aims such as to improve patient safety, implement evidence-based practices, address changing population needs or redirect resources to more pressing priorities [117].

This suggests that the construct of ‘disinvestment’ may be problematic in the local healthcare setting. After more than a decade of limited success, it may be time to consider new ways of approaching disinvestment. To stimulate research and debate, we propose two options that address some of the issues identified in this review; there may be others.

### Clarification and consolidation

This option proposes that the concept of ‘disinvestment’ as a specific aim and activity is clarified and consolidated from three perspectives.

Terminology: A common understanding of disinvestment between researchers and decision-makers with a single agreed definition and clear and consistent terminology to convey the underlying concepts would improve communication in disinvestment initiatives.

Research: Initiatives currently labelled as ‘disinvestment research’ are a mixed bag of activities. Several of these are well-established research fields in their own right, independent of disinvestment, for example HTA, PBMA, quality improvement and implementation science. In these situations the primary aim of the activity is not to disinvest; disinvestment is an outcome, by-product or part of the process. If there is to be a discipline of disinvestment research, it needs to be defined, theoretical underpinnings explored, and scope and methodologies agreed upon.

Application: Frameworks, models, methods and tools are needed. It has been proposed that mechanisms to develop, implement and evaluate disinvestment activities can be built on existing conceptual frameworks from other research paradigms such as HTA/HTR, PBMA, knowledge translation and implementation science [29, 123, 155]. As a step in this direction, an evidence-based framework for disinvestment in the context of resource allocation is proposed in Paper 10 in this series [84].

### Simplification and assimilation

This option proposes that disinvestment is considered as the opposite of investment; it is not a specific aim or activity, but is the outcome of, rather than the reason for, a resource allocation decision.

The definition is simplified. If investment is a process of allocating resources for the introduction, continuation or expansion of any aspect of the health system for any reason, disinvestment would be a process of withdrawing resources for the removal, reduction or restriction of any aspect of the health system for any reason. This makes the term more neutral by removing some of the emotive and negative connotations. Use of the term is likely to decrease as there is no need to use it to describe why or how cessation or limitation is being undertaken.

The approach is more constructive. Considering disinvestment within the spectrum of all resource allocation decisions [39, 50, 102, 103, 106, 117, 122] and from the perspective of optimising patient care and health outcomes [23, 34, 38, 39, 50, 123] is more positive and is closer to reality than undertaking disinvestment decisions and activities in isolation from other health service processes.

The activities are assimilated. The why and how of disinvestment embedded in the current definitions would be integrated within the language and methods and tools of familiar health service improvement paradigms such as EBHC, QI and system redesign.

There is still a need for research, development and application of methods to identify and address unsafe, ineffective, inefficient and inappropriate practices, but this does not need to be described as disinvestment, it can be achieved within the existing methodologies.

## Limitations

Although a rigorous systematic approach was taken to search the health databases and online publications (Additional file 1), it is impossible to be comprehensive in ascertaining all the relevant literature on disinvestment for the two reasons noted above.

Disinvestment in its broadest sense, cessation or limitation of something that was previously in practice, has always happened in health services but has not been labelled in this way. These decisions are mainly made and implemented in health care settings and, more recently, by government agencies. Neither of these groups typically publishes their work due to time pressures, competing priorities, lack of incentive to do so and, in the case of disinvestment, potential disincentives due to political sensitivities [26, 56].

The disinvestment literature is predominantly from developed countries and the generalisability to resource-poor settings may be limited.

These limitations mean that some relevant publications may not have been identified and some information has not been published. However, despite the limitations, several strong and consistent messages about disinvestment are evident. Unfortunately some of these consistent messages are about the lack of consistent messages.

The literature has been reviewed from the perspective of a local health service, however the authors’ experience is based in the Australian health system; hence differences with other health systems may not have been recognised and additional concepts or relationships may have been missed.

## Conclusions

Increasing use of new and existing health technologies and clinical practices has contributed to escalating costs and led to concerns about sustainability of the healthcare system. Some TCPs do not achieve the desired objectives and removing or restricting their use should improve health outcomes and reduce costs. While funders and health services have always made decisions about what is and is not provided, the construct of ‘disinvestment’ has emerged to describe the removal, reduction or restriction of current practices. The literature describes three main areas of opportunity for disinvestment: 1) TCPs in current use that were not evaluated rigorously prior to their introduction and have subsequently been identified as unsafe, ineffective or not cost-effective, 2) existing TCPs that are safe, effective and cost-effective but which have alternatives offering greater benefit and 3) TCPs that are overused or misused.

Early research and debate in disinvestment focused on national policy initiatives and local projects based on health economics approaches. Although the scope has widened considerably since, there is still little information to guide a systematic organisation-wide approach to disinvestment in the local healthcare context. The SHARE Program was established to address this.

There is no agreed terminology in this area. There are multiple definitions for disinvestment based on a range of different concepts, some overlap and others are mutually exclusive. There are also numerous alternative terms to convey the same concepts, some developed intentionally to avoid the negative connotations associated with the term disinvestment. Disinvestment is focused on the use of ‘health technologies’ but there is also a range of definitions for this term. To compound the difficulties in reaching a common understanding, the terms ‘disinvestment’ and ‘health technologies’ are used in one way by researchers and in another by decision-makers. Definitions of disinvestment are further complicated by constraints imposed by including a specified purpose (eg withdrawing practices of ‘low value’), defined criteria (eg effectiveness or cost-effectiveness) or anticipated outcome (eg reallocation of resources). This leaves no room for cessation of TCPs for other purposes, based on other criteria for different outcomes.

Investment is not defined in the health literature, but general use of the term reflects a process of allocating resources for the introduction, continuation or expansion of any aspect of the health system for any reason. Similarly, disinvestment could simply be ‘removal, reduction or restriction of any aspect of the health system for any reason’. Government and health service policy and guidance documents frequently use the phrase ‘investment and disinvestment’ without defining either term, indicating the continuum from funding to defunding or introduction to removal which represents the reality of decision-making. The various complex research definitions of disinvestment only capture fragments of this process. If this broad definition was used there would be no need to disinvest for the sake of disinvesting, and practice change would not be associated with the negatively-perceived purpose of ‘disinvestment’. Removal, reduction or restriction of existing practices would be driven by positive objectives such as reducing harm, improving outcomes, enhancing patient care, addressing national priorities, meeting local needs, introducing preferred alternatives, decreasing systematic errors and removing organisational waste. This approach is more likely to add incentives and reduce barriers to change.

Disinvestment is often undertaken in isolation from other decision-making systems and processes. Viewing disinvestment in the context of all resource allocation decisions with the purpose of optimising health care may also provide motivation for change, reduce disincentives and avoid some of the ethical dilemmas inherent in other disinvestment approaches.

Reinvestment is cited as a reason for and an outcome of disinvestment but there are no guarantees that resources will be released; costs may even increase. Health service accounting procedures and lack of data on usage of TCPs make it difficult to measure resources released from individual practice changes, and no reported methods for quantifying the resources released or reallocating them were identified.

There is considerable overlap between the aims, activities and outcomes of disinvestment initiatives and those of EBHC, QI, system redesign and PBMA. All of these are well-established in health service practice and research and have validated methods and tools. Given the negative connotations of disinvestment, and the lack of success in delivering projects which aimed ‘to disinvest’, perhaps removal, reduction and restriction of current practices would be more successful undertaken within existing healthcare paradigms.

We were unable to find any theories and found largely untested frameworks and models specifically for disinvestment. This is understandable given the variability and inconsistencies in terminology. Without common understanding of what ‘disinvestment’ is, the research agenda will continue to be a mixed bag of activities that belong to other domains. Researchers and decision-makers must reach agreement on definitions and concepts.

There is clearly a need to develop frameworks, models, methods and tools to systematically and proactively identify harmful, ineffective and inefficient TCPs, services and programs; to implement their removal, reduction or restriction; to evaluate the impact and outcomes of these changes; to measure savings if possible; and reallocate resources if appropriate. This can all be achieved without using the label ‘disinvestment’ which has been shown to have negative connotations and act as a barrier to change.

## Additional file

Additional file 1: Methods. (PDF 210 kb)

## Abbreviations

EBHC: Evidence based health care
HTA: Health technology assessment
HTR: Health technology reassessment
PBMA: Program budgeting and marginal analysis
QI: Quality improvement
SHARE: Sustainability in health care by allocating resources effectively
TCPs: Technologies and clinical practices
