# Supplementary material for: Sustainability in Health care by Allocating Resources Effectively (SHARE) 9: conceptualising disinvestment in the local healthcare setting
Source: BMC Health Serv Res. 2017 Sep 8;17:633. doi: 10.1186/s12913-017-2507-6 (PMC5591535; doi:10.1186/s12913-017-2507-6)
Supplement: Additional file 1: — Methods. (PDF 210 kb) [file 12913_2017_2507_MOESM1_ESM.pdf]

Sustainability in Health care by Allocating Resources Effectively (SHARE) 9: Conceptualising disinvestment in the local healthcare setting

Additional File: Methods

Papers 9 and 10 of the SHARE series present the findings of a review of the disinvestment literature, combined with findings from the SHARE Program, discussed from the perspective of the local healthcare setting.

The papers are presented as Debate rather than Research papers but, since the arguments are based on the findings of a literature review, readers need to have confidence that the process was rigorous and as comprehensive as possible.

Although undertaken systematically, this was not a systematic review. It is impossible to be absolutely comprehensive in ascertaining all the relevant literature on disinvestment for two main reasons.

- There is no general agreement about use of the term 'disinvestment', it is used to convey multiple concepts and there are many other terms used to convey the same range of concepts.
- The aims, activities and outcomes of disinvestment initiatives are replicated in research and practice in other healthcare paradigms and published in various bodies of literature.

Extensive searches were undertaken to ensure as much as possible that the discussion correctly reflects the literature.

Search strategy

General search

This search was undertaken at the commencement of the PhD (mid 2013) and was repeated half way through (mid 2015) and prior to completion of the final drafts of the two review papers (early 2016).

Sources: The initial search was conducted in medical databases (Ovid Medline, Ovid EMBASE, All EBM Reviews, The Cochrane Library) and the internet (via the Google search engine). Repeat searches were conducted in PubMed, The Cochrane Library and the internet via Google.

Search in PubMed: (((disinvest* OR decommission* OR defund* OR de-implement* OR de-adopt* OR "resource release" OR "resource allocation" OR reallocat* OR reinvest* OR "health technology reassessment")) AND ("1995/01/01"[PDat] : "3000/12/31"[PDat]) AND Humans[Mesh] AND English[lang]) Sort by: PublicationDate Filters: Publication date from 1995/01/01; Humans; English

Searches were adapted to suite the relevant databases.

Supplementary searches

Additional material was obtained from the following sources.

1. Health Technology Assessment International (HTAi) Disinvestment Special Interest Group regular email bulletins containing searches for disinvestment literature by Leigh-Ann Topfer, University of Alberta Health Technology and Policy Unit librarian.

   Search in PubMed: ((unnecessary procedures[mh]) OR (disinvest*[tiab]) OR ("low value"[ti]) OR ("choosing wisely"[tiab])) OR (((health care rationing[mh]) OR (cost control[mh]) OR (health priorities/ec) OR (resource allocation[mh]) OR (technology assessment, biomedical[mh]) OR (biomedical technology/ec) OR (budgets[mh]) OR (investments[mh]) OR (delivery of health care/ec) OR (cost-benefit analysis[mh]) OR (diffusion of innovation[mh]) OR (formularies as topic[mh]) OR (reimbursement mechanisms[mh]) OR (comparative effectiveness research[mh]) OR (decision making[mh]) OR (decision making, organizational[mh]) OR (priority setting[ti]) OR (coverage[ti]) OR (technology[ti]) OR (technologies[ti]) OR ("cost containment"[ti]) OR (rationing[ti]) OR (decision*[ti]) OR (reimburs*[ti]) OR (pmba[ti]) OR ("program budgeting" OR "programme budgeting") OR ("marginal analysis")) AND ((("de-adopt*") OR (deadopt*) OR (delist*[tiab]) OR ("de-list*"[tiab]) OR (ineffective[ti]) OR (redeploy*[ti]) OR (divest*[ti]) OR (obsolete[ti]) OR (obsolescen*[ti]) OR (abandon*[ti]) OR (decommission*[ti]) OR ("de-implement*") OR (deimplement*) OR ("phase out"[ti]) OR ("phasing out"[ti]) OR (reinvest[ti]) OR (reinvesting[ti]) OR (reinvestment[ti]) OR (discard*[ti]) OR (reassess*[ti]) OR (reallocat*[ti]) OR (disuse[ti]) OR (unnecessary[ti])) OR (defund*)))
2. Table of Contents of the International Journal of Technology Assessment in Health Care in regular email bulletins
3. Reference lists of relevant publications

Targeted searches

The need for additional searches was identified when it became apparent that

- there was no agreed definition for the term 'disinvestment', that it was used to convey multiple concepts, and that there were many other terms conveying the same range of concepts
- the aims, activities and outcomes of disinvestment initiatives were replicated in research and practice in other healthcare paradigms and published in various bodies of literature

Targeted searches were undertaken in the following situations to capture publications which would not have been identified using the specified disinvestment-related search terms but which were relevant to points under discussion in the reviews.

1. To further explore concepts within the identified themes. Some examples include definitions for health technologies; 'optimisation'; 'Choosing Wisely'; rationale for disinvestment; examples of theories, frameworks and models for disinvestment, resource allocation, prioritisation, etc; individual challenges specific to disinvestment; principles for disinvestment; methods and tools; barriers and enablers.
2. To capture information related to removal, reduction or restriction of health technologies and clinical practices that had not been labelled with a disinvestment-related term; such as publications in the EBP or quality and safety literature.
3. To ensure that no publications had been missed prior to making statements such as 'we were unable to find….'

Inclusion criteria

English language publications including guidelines, reviews, research studies, case studies, debate or discussion papers, technical reports or policy documents that addressed disinvestment were included.

Data extraction and analysis

No critical appraisal was undertaken as only a small proportion of included publications were research studies.

After the first search was conducted, full text versions of relevant papers were obtained.

Thematic analysis was undertaken from the perspective of the local healthcare setting [1].

- Familiarising with data: papers were read and notes taken.
- Generating initial codes: relevant features of the data were systematically coded and collated.
- Searching for themes: codes were collated into potential themes.
- Reviewing themes: themes were reviewed against the entire data set and a thematic map was developed.
- Defining and naming themes: the specifics of each theme and their place in the overall story were defined

All papers were re-read and any additional information related to the final themes that had been missed in the first reading was extracted.

Further information related to the final themes was extracted from additional publications subsequently identified by repeats of the general search and the supplementary and targeted searches.

Synthesis

The emergent themes could be categorised into two higher level themes, conceptualisation and operationalisation of disinvestment, which were used as the basis for two debate papers. The emergent themes were allocated between the two papers.

Information from the literature is considered from the perspective of the local healthcare setting and summarised narratively within each theme. This is combined with a narrative summary of findings from the SHARE Program relevant to the theme.

1. Vaismoradi M, Turunen H, Bondas T. Content analysis and thematic analysis: Implications for conducting a qualitative descriptive study. Nursing & health sciences. 2013;15(3):398-405. doi:10.1111/nhs.12048.
